# Risk factors for medication non-adherence among atrial fibrillation patients

**DOI:** 10.1186/s12872-019-1019-1

**Published:** 2019-02-11

**Authors:** Stephanie R. Reading, Mary Helen Black, Daniel E. Singer, Alan S. Go, Margaret C. Fang, Natalia Udaltsova, Teresa N. Harrison, Rong X. Wei, In-Lu Amy Liu, Kristi Reynolds, for the ATRIA-CVRN Investigators

**Affiliations:** 1 Department of Research and Evaluation, Kaiser Permanente Southern California, 100 S. Los Robles Ave., 2nd floor, Pasadena, CA 91101 USA; 2 Department of General Internal Medicine, Massachusetts General Hospital, Boston, MA USA; 3 Division of Research, Kaiser Permanente Northern California, Oakland, CA USA; 4 Departments of Epidemiology, Biostatistics and Medicine, University of California San Francisco, San Francisco, CA USA; 5 Division of Hospital Medicine, University of California San Francisco, San Francisco, CA USA

**Keywords:** Adherence, Epidemiology, Anticoagulants, Warfarin, Cardiology

## Abstract

**Background:**

Atrial fibrillation (AF) patients are routinely prescribed medications to prevent and treat complications, including those from common co-occurring comorbidities. However, adherence to such medications may be suboptimal. Therefore, we sought to identify risk factors for general medication non-adherence in a population of patients with atrial fibrillation.

**Methods:**

Data were collected from a large, ethnically-diverse cohort of Kaiser Permanente Northern and Southern California adult members with incident diagnosed AF between January 1, 2006 and June 30, 2009. Self-reported questionnaires were completed between May 1, 2010 and September 30, 2010, assessing patient socio-demographics, health behaviors, health status, medical history and medication adherence. Medication adherence was assessed using a previously validated 3-item questionnaire. Medication non-adherence was defined as either taking medication(s) as the doctor prescribed 75% of the time or less, or forgetting or choosing to skip one or more medication(s) once per week or more. Electronic health records were used to obtain additional data on medical history. Multivariable logistic regression analyses examined the associations between patient characteristics and self-reported general medication adherence among patients with complete questionnaire data.

**Results:**

Among 12,159 patients with complete questionnaire data, 6.3% (*n* = 771) reported medication non-adherence. Minority race/ethnicity versus non-Hispanic white, not married/with partner versus married/with partner, physical inactivity versus physically active, alcohol use versus no alcohol use, any days of self-reported poor physical health, mental health and/or sleep quality in the past 30 days versus 0 days, memory decline versus no memory decline, inadequate versus adequate health literacy, low-dose aspirin use versus no low-dose aspirin use, and diabetes mellitus were associated with higher adjusted odds of non-adherence, whereas, ages 65–84 years versus < 65 years of age, a Charlson Comorbidity Index score ≥ 3 versus 0, and hypertension were associated with lower adjusted odds of non-adherence.

**Conclusions:**

Several potentially preventable and/or modifiable risk factors related to medication non-adherence and a few non-modifiable risk factors were identified. These risk factors should be considered when assessing medication adherence among patients diagnosed with AF.

## Background

Atrial fibrillation (AF) is the most common clinically-significant adult arrhythmia [1, 2]. In 2010, 5.2 million people in the United States were estimated to have AF and a projected 12.1 million people were expected to have AF by the year 2030 [3]. For individuals with AF, the most frequent cardiovascular complications include ischemic stroke, heart failure and sudden cardiac death [4]. To prevent such complications, medications, including anticoagulants, are prescribed to lower the risk of ischemic stroke and other arterial thromboembolisms [5]. Medications for heart rate control and/or medications for rhythm control are also routinely prescribed [6–8]. Nevertheless, adherence to such prescribed medications is suboptimal and can translate into an increased risk of treatment failure, hospitalizations and early mortality [9–15].

In addition to the potential occurrence of AF-specific complications, many patients with AF present with non-cardiovascular specific comorbidities [16–19]. One study reported that 98% of patients with AF had at least one comorbidity [16]. The most commonly reported non-cardiovascular comorbidity was urologic disorders (62%) followed by chronic pain (61%), respiratory (42%), gastrointestinal (41%), sleep (29%), psychiatric (28%), cancer (26%) and dermatologic (26%) conditions. However, little is known about medication adherence to the treatment of these comorbidities and limited data exist regarding predictors of AF-specific medication non-adherence [15, 20]. It may be that patients with AF present as a unique sub-population among those with cardiovascular disease. According to the American Heart Association, many people with AF don’t recognize the seriousness of their illness (> 65%) [21] and may be less adherent to their medication compared to those in the general cardiovascular disease population. Additionally, patients with AF may not experience symptoms [21], leading to potentially lower adherence to medication. Although these instances are specific to treatments for AF, these behaviors may carry over into adherence to their other medication regimens.

It has been suggested, in studies assessing AF-specific medication adherence, that younger age may be one possible risk factor for non-adherence [22], but the findings for gender [23, 24], socioeconomic status [22, 25] and comorbidities [26, 27] are mixed. Additionally, dementia, mental function and the complexity of the dosing regimen have been found to decrease medication adherence in patients with AF [20, 22, 28]. Identifying those AF patients at greatest risk for general medication non-adherence remains a difficult task. To address this knowledge gap, we evaluated the associations between select patient characteristics and self-reported medication adherence within a large, ethnically diverse population of adults with incident diagnosed AF to elucidate those patients who may be at greatest risk for medication non-adherence.

## Methods

### Setting

Kaiser Permanente Northern California (KPNC) and Kaiser Permanente Southern California (KPSC) are two integrated healthcare delivery systems that currently provide comprehensive care to over 8 million individuals throughout the state of California. These > 8 million individuals represent a socio-demographically diverse population that is highly characteristic of the statewide population [29–31]. Complete details of the healthcare services that these individuals receive are captured through structured administrative and clinical databases managed through the EpicCare system (Epic Systems, Verona, WI).

### Study population

**Fig. 1:**
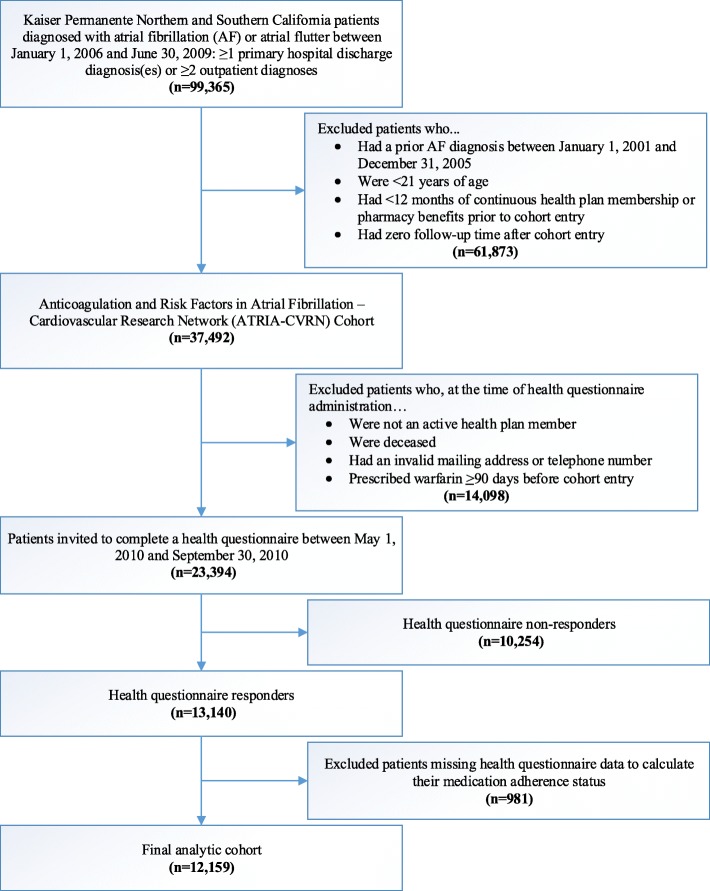
Assembly of the adult atrial fibrillation cohort with complete health questionnaire data to assess medication adherence status (*n* = 12,159)

The present investigation included KPNC and KPSC members who were part of the Anticoagulation and Risk Factors in Atrial Fibrillation – Cardiovascular Research Network (ATRIA-CVRN) cohort [32]. Details of this cohort have been previously described [1, 32–35]. In short, patients 21 years of age or older who were diagnosed with incident AF or atrial flutter between January 1, 2006 and June 30, 2009 were included (Fig. 1). Of these patients, a subset completed a 35-item health questionnaire. Questionnaire data were available from 13,140 patients with the median length of time between cohort entry and questionnaire completion being 2.65 years. Patients missing responses to the questions on medication adherence (*n* = 981) were excluded, leaving 12,159 patients for analysis. This study was approved by the ethics committees at Kaiser Permanente Northern and Southern California (reference numbers CN-09AGo-14-H and 5572, respectively). A waiver of written informed consent was obtained due to the nature of the study being a minimal-risk health questionnaire.

### Medication adherence

Medication adherence was assessed using three questions adapted from the Coronary Artery Risk Development in Young Adults (CARDIA) study [36]: (1) ‘In the past month, how often did you take your medications as the doctor prescribed?’ with the response options of ‘All of the time (100%)’, ‘Nearly all of the time (90%)’, ‘Most of the time (75%)’, ‘About half the time (50%)’ and ‘Less than half the time (<50%)’; (2) ‘In the past month, how often did you forget to take one or more of your prescribed medications?’ with the response options of ‘Never’, ‘Once’, ‘2–3 times’, ‘Once per week’, ‘Several times per week’ and ‘Nearly every day’; and (3) ‘In the past month, how often did you decide to skip one or more of your prescribed mediations’ with the same response options as question two. Medication non-adherence was defined as either (a) response to question one of “most of the time (75%)” or less, (b) response to question two of “once per week” or more, or (c) response to question three of “once per week” or more, based on previously validated definitions of non-adherence [37].

### Patient characteristics

Age and sex were determined from the patient’s electronic health record (EHR) at the time of AF diagnosis. Socio-demographic characteristics including race/ethnicity, marital status, educational attainment, and household income were obtained by questionnaire. Physical activity (based on frequency, duration and intensity of activity in the past month) and height, weight, cigarette, alcohol and aspirin use during the year prior were also obtained by questionnaire. Height and weight were used to calculate body mass index (BMI kg/m^2^). Self-reported health status was also collected from the questionnaire to evaluate frequency of poor physical and mental health in the past month prior to questionnaire completion (range: 0 to 30 days), overall current health (range: poor to excellent), if sleep was affecting daily function (range: never to almost every day), memory decline and health literacy (based on a validated 3-item instrument examining problems due to reading, understanding and completing medical forms dichotomized into adequate or inadequate [38–41]).

History of coronary heart disease, chronic heart failure, dementia, depression, diabetes mellitus, hypertension, ischemic stroke and transient ischemic attack were extracted from the EHRs using ICD-9-CM codes obtained from both inpatient and outpatient encounters during the 5-year period prior to questionnaire completion. These data were used to calculate a CHADS_2_ stroke risk score [26]. A Charlson Comorbidity Index (CCI) score was also calculated using ICD-9-CM codes from both inpatient and outpatient encounters during the 1-year period prior to questionnaire completion, to account for overall comorbidity burden and risk of mortality [42].

### Statistical analysis

Socio-demographic characteristics, health behaviors, self-reported health status and medical history were compared across medication adherence status using chi-square, Fisher’s exact and Wilcoxon signed-rank tests, as appropriate. Adjusted odds ratios (ORs) and 95% confidence intervals (CIs) for non-adherence were estimated using multivariable logistic regression models among all patients with complete questionnaire data, with all patient characteristics available in the survey included as candidate predictors. All analyses were conducted using SAS software version 9.2 (SAS Institute, Cary, NC).

## Results

Of the 12,159 patients with incident diagnosed AF who responded to the questionnaire items regarding medication adherence, 771 (6.3%) were categorized as not adherent to their prescribed medications (Table 1). Patients who self-reported non-adherence to prescribed medications were younger (< 65 years of age) and more likely to be a racial/ethnic minority, not married or living with a partner and have a household income of $25,000 or less compared to those who were adherent (Table 2). Non-adherent patients were also more likely to report less physical activity, be a current smoker and consume alcohol. In addition, non-adherent patients were more likely to self-report more than 1 day of poor physical and/or mental health in the last month, have fair or poor current overall health, indicate that sleep affected their daily function more than 1 day a week, have memory decline in the past 2–3 years and have inadequate health literacy compared to those patients who were adherent. Lastly, non-adherent patients were less likely to have hypertension, but more likely to report low-dose aspirin use in the last year, have a BMI ≥30, CHADS_2_ score of 0, depression and diabetes mellitus compared to adherent patients. Most univariate associations were retained in the multivariable model, with a few exceptions (Table 3). In particular, household income, cigarette use, self-rated current health, BMI and depression status were not statistically significant independent correlates of medication non-adherence. However, having a CCI score ≥ 3 was associated with a decreased adjusted odds of medication non-adherence, after accounting for other patient characteristics.

**Table 1:** Responses to medication adherence questions from 12,159 incident atrial fibrillation patients

	n (%)
Question	
1. In the past month, how often did you take your medications as the doctor prescribed?	
All of the time (100%)	10,120 (83.2)
Nearly all of the time (90%)	1726 (14.2)
Most of the time (75%)	202 (1.7)
About half the time (50%)	37 (0.3)
Less than half the time (<50%)	74 (0.6)
2. In the past month, how often did you forget to take one or more prescribed medications?	
Never	7147 (58.8)
Once	3061 (25.2)
2–3 times	1551 (12.8)
Once per week	281 (2.3)
Several times per week	73 (0.6)
Nearly every day	46 (0.4)
3. In the past month, how often did you decide to skip one or more prescribed medications?	
Never	10,599 (87.2)
Once	734 (6.0)
2–3 times	505 (4.2)
Once per week	117 (1.0)
Several times per week	96 (0.8)
Nearly every day	108 (0.9)
Medication Adherence	
Adherent	11,388 (93.7)
Not Adherent^a^	771 (6.3)

^a^Not adherent defined as (a) an answer to question 1 as “most of the time (75%)” or less or (b) an answer to question 2 as “once per week” or more or (c) an answer to question 3 as “once per week” or more

**Table 2:** Characteristics of 12,159 incident atrial fibrillation patients by medication adherence status

	Adherent *n* = 11,388 (93.7%)	Not Adherent *n* = 771 (6.3%)	P^†^
Socio-demographics			
Age, years			< 0.001
Median	72.7	70.1	
25th – 75th %	64.4–79.9	59.5–79.1	
Age group, years, n (%)			< 0.001
< 65	3017 (26.5)	293 (38.0)	
65–74	3549 (31.2)	198 (25.7)	
75–84	3523 (30.9)	185 (24.0)	
≥ 85	1299 (11.4)	95 (12.3)	
Male, n (%)	6498 (57.1)	435 (56.4)	0.735
Race/Ethnicity, n (%)			< 0.001
Non-Hispanic White	8505 (74.7)	502 (65.1)	
Non-Hispanic Black	572 (5.0)	81 (10.5)	
Non-Hispanic Asian/Pacific Islander	775 (6.8)	64 (8.3)	
Hispanic	889 (7.8)	75 (9.7)	
Other/Unknown	647 (5.7)	49 (6.4)	
Marital Status, n (%)			0.006
Married/Partner	7470 (65.6)	463 (60.1)	
Not Married/Partner	3788 (33.3)	300 (38.9)	
Unknown	130 (1.1)	8 (1.0)	
Educational Attainment, n (%)			0.299
Less than High School	929 (8.2)	72 (9.3)	
High School Graduate	2110 (18.5)	145 (18.8)	
Some College	4075 (35.8)	290 (37.6)	
Bachelor’s Degree or Higher	3999 (35.1)	243 (31.5)	
Unknown	275 (2.4)	21 (2.7)	
Household Income, n (%)			0.008
$25,000 or less	1694 (14.9)	150 (19.5)	
$25,001 – 50,000	2477 (21.8)	168 (21.8)	
$50,001 – 80,000	1967 (17.3)	122 (15.8)	
More than $80,000	2501 (22.0)	169 (21.9)	
Unknown	2749 (24.1)	162 (21.0)	
Questionnaire Language, n (%)			0.349
English	11,077 (97.3)	752 (97.5)	
Spanish	280 (2.5)	19 (2.5)	
Mandarin	31 (0.3)	0 (0.0)	
Region, n (%)			0.941
Northern California	5854 (51.4)	395 (51.2)	
Southern California	5534 (48.6)	376 (48.8)	
Health Behaviors			
Physical Activity (past month), n (%)			< 0.001
None	854 (7.5)	86 (11.2)	
Low/Moderate	7683 (67.5)	532 (69.0)	
High	2802 (24.6)	149 (19.3)	
Unknown	49 (0.4)	4 (0.5)	
Cigarette Use (past year), n (%)			0.002
Never	4758 (41.8)	303 (39.3)	
Former	5759 (50.6)	383 (49.7)	
Current	624 (5.5)	67 (8.7)	
Unknown	247 (2.2)	18 (2.3)	
Alcohol Use (past year), n (%)			< 0.001
Never	2364 (20.8)	105 (13.6)	
Former	2245 (19.7)	169 (21.9)	
Current	6585 (57.8)	484 (62.8)	
Unknown	194 (1.7)	13 (1.7)	
Self-Reported Health Status			
Poor Physical Health (past month), n (%)			< 0.001
0 days	6313 (55.4)	320 (41.5)	
1–7 days	2156 (18.9)	196 (25.4)	
8–21 days	816 (7.2)	78 (10.1)	
22–28 days	80 (0.7)	7 (0.9)	
≥ 29 days	821 (7.2)	74 (9.6)	
Unknown	1202 (10.6)	96 (12.5)	
Poor Mental Health (past month), n (%)			< 0.001
0 days	7835 (68.8)	400 (51.9)	
1–7 days	1401 (12.3)	134 (17.4)	
8–21 days	593 (5.2)	66 (8.6)	
22–28 days	66 (0.6)	6 (0.8)	
≥ 29 days	422 (3.7)	50 (6.5)	
Unknown	1071 (9.4)	115 (14.9)	
Self-Rated Current Health, n (%)			< 0.001
Poor	641 (5.6)	59 (7.7)	
Fair	2581 (22.7)	228 (29.6)	
Good	4561 (40.1)	271 (35.2)	
Very Good	2890 (25.4)	168 (21.8)	
Excellent	625 (5.5)	36 (4.7)	
Unknown	90 (0.8)	9 (1.2)	
Sleep Affecting Daily Function (average), n (%)			< 0.001
Never	2747 (24.1)	145 (18.8)	
Rarely	5283 (46.4)	294 (38.1)	
1–3 days/week	1685 (14.8)	154 (20.0)	
4–6 days/week	431 (3.8)	44 (5.7)	
Almost every day	1060 (9.3)	126 (16.3)	
Unknown	182 (1.6)	8 (1.0)	
Memory Decline (past 2–3 years), n (%)			< 0.001
No	5582 (49.0)	294 (38.1)	
Yes	4601 (40.4)	397 (51.5)	
Unknown	1205 (10.6)	80 (10.4)	
Health Literacy, n (%)			< 0.001
Adequate	8813 (77.4)	536 (69.5)	
Inadequate	2575 (22.6)	235 (30.5)	
Medical History			
Low-Dose Aspirin (past year; ≤100 mg/tablet), n (%)			0.043
Did not use in the last 12 mo.	6462 (56.7)	404 (52.4)	
Did use in the last 12 mo.	4488 (39.4)	339 (44.0)	
Unknown	438 (3.9)	28 (3.6)	
Aspirin or Aspirin-Product (past year; ≥325 mg/tablet), n (%)			0.240
Did not use in the last 12 mo.	8431 (74.0)	551 (71.5)	
Did use in the last 12 mo.	2287 (20.1)	174 (22.6)	
Unknown	670 (5.9)	46 (6.0)	
Body Mass Index (kg/m^2^), n (%)			0.003
< 25	3211 (28.2)	191 (24.8)	
25–30	3915 (34.4)	254 (32.9)	
≥ 30	3492 (30.7)	284 (36.8)	
Unknown	770 (6.8)	42 (5.5)	
Charlson Comorbidity Index (past year), n (%)			0.124
0	3773 (33.1)	255 (33.1)	
1	2346 (20.6)	175 (22.7)	
2	1652 (14.5)	124 (16.1)	
≥ 3	3617 (31.8)	217 (28.2)	
CHADS_2_ Score (past five years), n (%)			0.049
0	1849 (16.2)	155 (20.1)	
1	3959 (34.8)	254 (32.9)	
2	3782 (33.2)	245 (31.8)	
≥ 3	1798 (15.8)	117 (15.2)	
Coronary Heart Disease, n (%)	1372 (12.1)	92 (11.9)	0.955
Chronic Heart Failure, n (%)	2457 (21.6)	167 (21.7)	0.964
Dementia, n (%)	288 (2.5)	19 (2.5)	> 0.999
Depression, n (%)	2309 (20.3)	191 (24.8)	0.004
Diabetes Mellitus, n (%)	3096 (27.2)	236 (30.6)	0.041
Hypertension, n (%)	8961 (78.7)	568 (73.7)	0.001
Ischemic Stroke or Transient Ischemic Attack, n (%)	604 (5.3)	39 (5.1)	0.868

^**†**^*p*-values based on chi-square, Fisher’s exact and Wilcoxon signed-rank tests

**Table 3:** Adjusted odds ratios (95% confidence intervals) predicting medication non-adherence among 12,159 atrial fibrillation patients

	Atrial Fibrillation Patients (n = 12,159)
Socio-demographics	
Age group, years	
< 65	REF
65–74	0.68 (0.55, 0.83)^a^
75–84	0.67 (0.53, 0.84)^a^
≥ 85	0.86 (0.64, 1.16)
Gender	
Male	REF
Female	0.97 (0.82, 1.15)
Race/Ethnicity	
Non-Hispanic White	REF
Non-Hispanic Black	2.26 (1.73, 2.96)^a^
Non-Hispanic Asian/Pacific Islander	1.59 (1.19, 2.13)^b^
Hispanic	1.29 (0.98, 1.70)
Other/Unknown	1.24 (0.89, 1.71)
Marital Status	
Married/Partner	REF
Not Married/Partner	1.24 (1.05, 1.48)^c^
Unknown	1.24 (0.53, 2.90)
Educational Attainment	
Less than High School	1.07 (0.78, 1.48)
High School Graduate	1.01 (0.80, 1.29)
Some College	1.07 (0.88, 1.29)
Bachelor’s Degree or Higher	REF
Unknown	1.35 (0.81, 2.27)
Household Income	
$25,000 or less	1.17 (0.89, 1.55)
$25,001 – 50,000	1.00 (0.78, 1.28)
$50,001 – 80,000	0.94 (0.73, 1.21)
More than $80,000	REF
Unknown	0.95 (0.74, 1.22)
Health Behaviors	
Physical Activity (past month)	
None	1.57 (1.16, 2.13)^b^
Low/Moderate	1.13 (0.93, 1.38)
High	REF
Unknown	1.81 (0.61, 5.37)
Cigarette Use (past year)	
Never	REF
Former	0.98 (0.83, 1.16)
Current	1.24 (0.92, 1.67)
Unknown	1.00 (0.59, 1.70)
Alcohol Use (past year)	
Never	REF
Former	1.69 (1.30, 2.20)^a^
Current	1.91 (1.51, 2.43)^a^
Unknown	1.36 (0.72, 2.58)
Self-Reported Health Status	
Poor Physical Health (past month)	
0 days	REF
1–7 days	1.43 (1.17, 1.75)^a^
8–21 days	1.28 (0.96, 1.72)
22–28 days	1.08 (0.47, 2.46)
≥ 29 days	1.17 (0.84, 1.61)
Unknown	1.10 (0.83, 1.46)
Poor Mental Health (past month)	
0 days	REF
1–7 days	1.31 (1.05, 1.63)^c^
8–21 days	1.35 (0.99, 1.83)
22–28 days	0.96 (0.40, 2.34)
≥ 29 days	1.44 (1.00, 2.06)^c^
Unknown	1.78 (1.38, 2.31)^a^
Self-Rated Current Health	
Poor	0.80 (0.49, 1.32)
Fair	0.94 (0.63, 1.40)
Good	0.79 (0.54, 1.15)
Very Good	0.94 (0.64, 1.37)
Excellent	REF
Unknown	1.20 (0.54, 2.69)
Sleep Affecting Daily Function (average)	
Never	REF
Rarely	1.01 (0.82, 1.25)
1–3 days/week	1.35 (1.05, 1.73)^c^
4–6 days/week	1.46 (1.01, 2.11)^c^
Almost every day	1.56 (1.19, 2.04)^b^
Unknown	0.55 (0.24, 1.26)
Memory Decline (past 2–3 years)	
No	REF
Yes	1.34 (1.13, 1.59)^a^
Unknown	1.16 (0.89, 1.51)
Health Literacy	
Adequate	REF
Inadequate	1.32 (1.09, 1.60)^b^
Medical History	
Baby or Low-Dose Aspirin (past year; ≤100 mg/tablet)	
Did not use in the last 12 mo.	REF
Did use in the last 12 mo.	1.21 (1.03, 1.42)^c^
Unknown	1.01 (0.66, 1.56)
Aspirin or Aspirin-Product (past year; ≥325 mg/tablet)	
Did not use in the last 12 mo.	REF
Did use in the last 12 mo.	1.03 (0.85, 1.25)
Unknown	0.90 (0.64, 1.26)
Body Mass Index (kg/m^2^)	
< 25	REF
25–30	1.07 (0.87, 1.31)
≥ 30	1.20 (0.96, 1.48)
Unknown	0.87 (0.61, 1.24)
Charlson Comorbidity Index (past year)	
0	REF
1	1.06 (0.86, 1.31)
2	1.08 (0.84, 1.38)
≥ 3	0.77 (0.60, 1.00)^c^
Coronary Heart Disease	
No	REF
Yes	1.01 (0.79, 1.28)
Chronic Heart Failure	
No	REF
Yes	0.98 (0.80, 1.20)
Dementia	
No	REF
Yes	0.76 (0.46, 1.24)
Depression	
No	REF
Yes	0.96 (0.80, 1.16)
Diabetes Mellitus	
No	REF
Yes	1.22 (1.01, 1.48)^c^
Hypertension	
No	REF
Yes	0.72 (0.60, 0.87)^a^
Ischemic Stroke or Transient Ischemic Attack	
No	REF
Yes	0.96 (0.68, 1.35)

^a^ < 0.001

^b^ < 0.01

^c^ < 0.05

## Discussion

Among a large, ethnically-diverse sample of adults with incident AF receiving medical care within an integrated healthcare delivery system, we classified 6.3% as being not optimally adherent to prescribed medications per self-report. Patients were more likely to be non-adherent to their prescribed medications if they were a racial/ethnic minority versus non-Hispanic white, not married/with partner, physical inactive, used alcohol, had any days of self-reported poor physical health, mental health and/or sleep quality in the past 30 days versus 0 days, had memory decline, inadequate health literacy, low-dose aspirin use and/or diabetes mellitus. Whereas, patients who were of older age (65–84 years versus < 65 years of age), had a Charlson Comorbidity Index score ≥ 3 versus 0 and had hypertension were less likely to be non-adherent.

Several studies have estimated one-year non-adherence rates to oral anticoagulants among AF patients to be 3–28% based on the type of oral anticoagulant prescribed [11–13, 43]. Although our study assessed general medication adherence, which included assessment of both medication adherence to AF-specific treatment and any medication used to treat comorbidities, our medication non-adherence rate within our full AF cohort (6.3%) was on the lower end of what has been previously estimated in an AF population. More broadly, it has been shown that self-reported medication adherence to cardiovascular disease medications is less than 40% [44] and in an elderly population with a range of chronic illnesses only 45% had good medication adherence [45]. Our finding of only 6.3% medication non-adherence may be due, in part, to our cohort receiving care within an integrated healthcare delivery system that emphasizes coordination of care across different providers and clinical setting through a single EHR, as opposed to the more fragmented care seen in other types of healthcare delivery systems [46, 47]. Additionally, our assessment of medication adherence was based on self-report. This may have introduced some recall, social desirability and/or interviewer bias, leading to an over-estimate of the patient’s actual medication adherence [48]. Nonetheless, using this brief self-reported measure of medication adherence increased our ability to capture adherence information from a larger population (in our case 12,159 atrial fibrillation patients) due to the ease and cost-effectiveness of implementation in a clinical setting [49]. Additionally, self-reported medication adherence measures may also have the benefit of demonstrating high specificity in capturing people who are truly non-adherent [48, 49]. This was particularly valuable in our analyses, where we aimed to better understand population-level patient factors that may be associated with medication non-adherence in patients with atrial fibrillation. Self-reported adherence measures have also been shown to correlate well with adherence captured by pill counts and other monitoring devices [50], as well as with pharmacy dispensing records [51].

Results from the univariate analyses showed that patients who self-reported non-adherence to prescribed medications generally reported worse health indicators, as well as, factors related to low socio-economic status (racial/ethnic minority, being unmarried, lower household income, physical inactivity, BMI ≥30, alcohol and cigarette use, poor physical, mental and current health, decreased sleep quality, memory decline, inadequate health literacy and both depression and diabetes mellitus) which are similar to those factors that have been reported with non-adherence in other chronic conditions [52]. Many of these risk factors for medication non-adherence may also be preventable and/or modifiable. Physical inactivity and alcohol use, although most likely not directly related to medication adherence, can lead to poor physical health, mental health and sleep quality. These later three factors have all been associated with medication non-adherence across populations [53, 54]. Poor health literacy, on the other hand, may be both modifiable and preventable. It is also one of the most common problems associated with medication non-adherence [55]. Additionally, our study found that non-adherent patients were less likely to have hypertension, but more likely to report low-dose aspirin use in the last year and have CHADS_2_ score of 0 when compared to patients who were adherent. The finding that patients who had hypertension were more likely to be adherent to their medication(s) is most likely due to the success of the Kaiser Permanente Hypertension Control Program [56]. From 2001 to 2013, hypertension control within KPNC increased from 44 to 90% [57]. One aspect of this program encouraged single pill combination therapy — combining multiple drugs into one pill. This strategy improved adherence, lowered patient costs and improved blood pressure control. Due to the program’s success, Kaiser Permanente Southern California also implemented these strategies into their region. Thus, many, if not all, patients included in our study were recipients of this program.

In our multivariable regression analysis, results paralleled those from the univariate associations. Of note, however, was the finding that higher CCI score (≥3) was linked to lower adjusted odds of being non-adherent compared to a CCI score of 0. These results may reflect the impact of increased contact with healthcare providers, which can improve medication adherence accountability [46, 47].

Considering these findings, we also acknowledge that the cross-sectional and observational nature of these data precluded us from assessing longitudinal trends in medication adherence among our patients with incident AF. Additionally, we were unable to assess polypharmacy for inclusion in our adjusted models. Thus, it may be that our low non-adherence rate was influenced by the relatively few prescriptions prescribed per patient. We were also unable to measure time since diagnosis to questionnaire completion. As adherence rates to AF-specific medications have been shown to decline with time, it may be that many of our patients completed the questionnaire soon after being diagnosed with atrial fibrillation. Thus, adherence for those patients could have been misleadingly high. However, a strength of our study was that we had access to a large, socio-demographically diverse population of validated incident diagnosed AF patients. This allowed for the detailed investigation into the relationships between a wide range patient factors and medication adherence status within a highly representative population of AF patients.

## Conclusions

In an ethnically-diverse cohort of AF patients, we identified multiple possible risk factors for medication non-adherence. These include selected socio-demographic characteristics, lifestyle factors, self-reported poor physical health, mental health and/or sleep quality, having memory decline, inadequate health literacy, using low-dose aspirin, having diabetes mellitus and a higher comorbidity burden. This has broad implications for both patients and providers when managing care for patients with AF, where maximizing the benefit from AF medication and treatment for any existing comorbidities involves understanding the patient factors associated with medication non-adherence. Additionally, by finding ways to intervene on those risk factors that may be preventable and/or modifiable may improve overall medication adherence and bring awareness to those patients at highest risk for non-adherence.

## Abbreviations

AF: Atrial fibrillation
ATRIA-CVRN: Anticoagulation and Risk Factors in Atrial Fibrillation – Cardiovascular Research Network
BMI: Body mass index
CARDIA: Coronary Artery Risk Development in Young Adults
CCI: Charlson Comorbidity Index
CI: Confidence interval
DOACs: Direct-acting oral anticoagulants
ECG: Electrocardiogram
EHR: Electronic health record
ICD-9-CM: International Classification of Diseases, Ninth Revision, Clinical Modification
KPNC: Kaiser Permanente Northern California
KPSC: Kaiser Permanente Southern California
OR: Odds ratio
